# Establishment of a prediction model for long-term infections in patients undergoing peritoneal dialysis

**DOI:** 10.3389/fmed.2025.1596403

**Published:** 2025-06-17

**Authors:** Mengmeng Liu, Qian Lu, Jinzhu Sun, Shengnan Dai, Feng Yan, Chun Yang, Qiuhua Zhang

**Affiliations:** ^1^Department of Nephrology, Wuxi No.2 People’s Hospital, Jiangnan University Medical Center, Wuxi, China; ^2^Department of Urology, Wuxi No.2 People’s Hospital, Jiangnan University Medical Center, Wuxi, China

**Keywords:** end-stage renal disease, peritoneal dialysis, long-term infection, prediction model, nomogram

## Abstract

**Background and Objective:**

Peritoneal dialysis (PD)-associated infections are the primary contributors to PD technique failure and patient mortality. Given these reasons, this study aims to identify independent risk factors for long-term infections in PD individuals and to construct an effective clinical prediction model using multivariate analysis.

**Methods:**

This study retrospectively analyzed 214 participants with ESRD who underwent PD catheterization at Wuxi No. 2 People’s Hospital. Based on whether they developed infections or not after 3 months treatment of regular peritoneal dialysis, all patients were categorized into two cohorts: infected (*n* = 67) and non-infected (*n* = 147). A comparison of clinical indicators was made between the two cohorts, and independent risk factors were initially determined through the means of univariate and multivariate logistic regression analyses for infections in PD patients. Via R software, we constructed a nomogram prediction model, its performance was validated.

**Results:**

Age (*p* = 0.004), surgical incision length (*p* = 0.018), and Prognostic Nutritional Index (PNI,* p* < 0.001) were identified as independent risk factors for long-term infections in PD patients. Based on the three significant predictors, we constructed a nomogram model, of which predictive performance was assessed through analysis of the ROC curve, which revealed area under the curve (AUC) values of 0.807, demonstrating good discriminative ability of the prediction model for long-term infection risk in PD patients.

**Conclusion:**

Advanced age, lower PNI, and longer surgical incision length are closely linked to the occurrence of infections in PD individuals. The nomogram model which was based on this study showed high efficacy in predicting long-term infections and can serve as a reference to recognize individuals with a high likelihood of complications for medical caregivers as early as possible.

## 1 Introduction

Recent evidence suggests that there has been a rapid growth in the number of people with ESRD in China, resulting in a significant rise in total count of patients undergoing dialysis (1). By the end of 2023, China was reported to have the highest total of individuals receiving dialysis globally, with over 1.06 million registered for either hemodialysis or peritoneal dialysis. Between 2013 and 2023, the number of patients undergoing PD increased by 108,000. PD accounts for approximately 11% of dialysis cases globally and is a key renal replacement therapy for ESRD patients (2, 3). PD utilizes the semipermeable nature of the peritoneum to remove toxins and metabolic waste through diffusion and convection, thereby achieving blood purification (4, 5). Compared to hemodialysis, PD offers several advantages, including the convenience of home treatment, ease of operation, supported conservation of remaining renal function, and safety (6). As a result, it is widely recognized and promoted by numerous experts in the field. However, studies indicate that patients with ESRD have no less than 50% probability of experiencing one or more infections during dialysis (7), which are among the leading causes of discontinuation of dialysis treatment and increased mortality–second only to cardiovascular disease. Infections related to PD are a major factor influencing prognosis (8). Predicting and identifying patients at high risk of PD-related infections, followed by timely intervention, is essential for improving survival rates, quality of life, and overall outcomes. We aimed to develop a high-performing model which is able to help predict the underlying risk elements of long-term infections in patients undergoing PD using advanced statistical methods and big data investigation.

## 2 Materials and methods

### 2.1 Study design and participants

214 participants with ESRD who underwent PD catheterization and received regular PD treatment for over 3 months at Wuxi No. 2 People’s Hospital from June 2017 to June 2024 were parts of this study. The primary endpoint of our study is the occurrence of infections in PD patients during the observation period from June 2017 to June 2024. Based on patients’ infection status after 3 months of PD, they were categorized into an infection group and a non-infection one. Infections primarily included intra-abdominal, skin, and catheter-related infections (tunnel and exit-site infections). The Ethics Committee of Wuxi No. 2 People’s Hospital (Ethics Approval No.2023Y-3) issued strong approval for the ethical conduct of this study. Inclusion criteria: (1) diagnosed with ESRD based on a glomerular filtration rateless than 15 ml/(min.1.73 m^2^), (2) treated with PD following the “Chinese Guidelines for Peritoneal Dialysis Catheterization” with a treatment duration of ≥ 3 months (9), (3) aged 18–85 years, (4) complete clinical laboratory and imaging examination data available, (5) signed informed consent. Exclusion criteria: (1) lack of regular follow-up at the hospital, (2) severe cardiopulmonary dysfunction, (3) severe intra-abdominal infections (occurring after PD catheter placement surgery but before the initiation of the first PD treatment) or adhesions, (4) pregnancy, and (5) failure to sign informed consent.

### 2.2 Data collection

At the time of admission, relevant examinations were conducted, and general data were collected. These comprised essential information (age, gender, weight, height, BMI), laboratory results (serum creatinine, blood urea nitrogen, serum sodium, serum potassium, albumin and other blood count results), surgical-related indicators (operative time, intraoperative blood loss, surgical incision length, postoperative hospitalization duration, surgery costs, surgery costs/total costs), and peritoneal dialysis-related complications (catheter flow dysfunction, dialysate leakage, catheter migration, exit-site infections, and abdominal catheter tunnel infection).

### 2.3 Nomogram for individualized prediction

We carried out multivariable logistic regression analysis with stepwise selection guided by the Akaike Information Criterion (AIC) to identify independent risk factors for infections in PD patients. Variables demonstrating statistical significance (*p* < 0.05) were retained as independent predictors. Random forest plotting was subsequently implemented to visualize predictor accuracy and variable importance metrics, which were ultimately integrated into a nomogram for predicting infection risk in PD patients. We plotted the ROC curve and evaluated the model’s discriminative ability by calculating the area under the curve (AUC). Additionally, calibration performance was quantified via 1,000-round bootstrap validation of calibration curves, while decision curve analysis (DCA) was carried out by calculating standardized net benefits across varying threshold probabilities to evaluate the clinical utility of the nomogram (10), which compensates for the limitations of ROC curves (11).

### 2.4 Statistical methods

We used SPSS 20.0 statistical to make an analysis of the classified and structured data. Continuous variables were expressed as mean ± standard deviation or median (interquartile range, IQR), depending on normality. We made use of *t*-test or Mann-Whitney U test to compare the means or medians across different sets. With respect to categorical variables, the data were presented in terms of frequency counts and percentage, at the same time, the implementation of Pearson’s chi-square test facilitated a comprehensive evaluation of the variations identified between the two groups. Odds ratios (ORs), often with 95% confidence intervals (CIs), were used to present the entire set of findings. Univariate analysis was performed using Pearson’s chi-square test or Fisher’s exact test, as appropriate. Variables with statistically significant differences (*p* < 0.05) were included in a multivariate logistic regression model (backward direction) to identify independent risk factors for long-term infection in PD patients. And validation of the nomogram were executed using R software (x64 for Windows, version 3.6.1). In all analyses, a two-sided *p*-value < 0.05 was considered statistically significant.

## 3 Result

### 3.1 Baseline characteristics of patients

**FIGURE 1:**
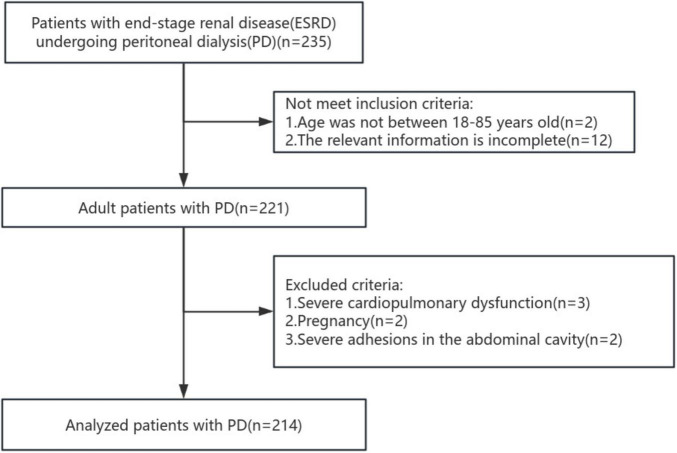
The flow chart of study design and patient selection.

235 individuals with ESRD who underwent PD were initially screened. Following the screening process, 214 participants satisfying the inclusion criteria were finally included. The procedural steps involved in the data selection process are depicted in the flowchart feature in Figure 1.

We collected clinical data from 214 patients with ESRD undergoing regular peritoneal dialysis. Based on the follow-up results after 3 months of PD treatment, all participants were stratified into infection and non-infection groups. Significant intergroup differences were observed in the following aspects: age, serum albumin, PNI, GNRI, surgery-related indicators (surgical duration, intraoperative bleeding volume, surgical incision length), primary disease and peritoneal dialysis-associated complications [exit-site infection (ESI) and abdominal catheter tunnel infection] (all with *p* < 0.05). Individuals in the infection group were older than those in the non-infection one (57.81 ± 11.95 years vs. 52.25 ± 13.29 years). Moreover, the infection group exhibited significantly longer surgical incision lengths (3.28 ± 0.93 cm vs. 2.83 ± 0.82 cm) and lower PNI values (37.04 ± 6.00 vs. 41.99 ± 4.97) compared to the non-infection group. In both groups, diabetic nephropathy was documented as the second most frequent primary disease, highlighting its substantial impact (12). There were no statistically significant intergroup differences in: hematologic indicators (serum creatinine, blood urea nitrogen, serum sodium, serum calcium, etc.), peritoneal dialysis-related complications (catheter malfunction, dialysate leakage, catheter migration). The clinical features of both groups are shown in Table 1.

**TABLE 1:** Baseline characteristics of patients.

Characteristics	No infection (*n* = 147)	Infection (*n* = 67)	*P*-value
Age, mean ± sd	52.25 ± 13.29	57.81 ± 11.95	0.004
Gender (Female 0, Male 1), *n* (%)			0.124
0	60.00 (40.80%)	20.00 (29.90%)	
1	87.00 (59.20%)	47.00 (70.10%)	
Height,cm, median (IQR)	167.00 (160.00, 173.00)	165.00 (160.00, 172.00)	0.630
Weight, median (IQR)	64.00 (56.50, 72.00)	62.50 (55.00, 73.50)	0.638
BMI, median (IQR)	22.66 (20.76, 25.07)	23.57 (21.22, 25.78)	0.170
Serum creatinine, mean ± sd	816.20 ± 333.27	839.06 ± 291.46	0.629
Blood urea nitrogen, mean ± sd	36.44 ± 47.96	32.28 ± 10.93	0.484
Serum sodium, mean ± sd	139.75 ± 3.17	139.79 ± 3.59	0.934
Serum potassium, mean ± sd	8.00 ± 42.61	4.59 ± 0.77	0.514
Serum calcium, mean ± sd	1.99 ± 0.26	1.98 ± 0.28	0.844
Serum phosphorus, mean ± sd	2.57 ± 8.43	1.93 ± 0.54	0.540
Serum albumin, mean ± sd	36.33 ± 4.72	31.77 ± 5.44	2.252e−09
Blood PTH, mean ± sd	428.16 ± 330.10	492.57 ± 455.78	0.247
Total cholesterol (mmol), mean ± sd	4.28 ± 1.31	4.14 ± 1.15	0.453
White blood cell count (× 10^9^), mean ± sd	6.46 ± 2.90	6.37 ± 1.92	0.815
Neutrophil count (10^9^), mean ± sd	4.64 ± 2.73	4.62 ± 1.73	0.957
Lymphocyte count (10^9^), mean ± sd	1.13 ± 0.50	1.05 ± 0.50	0.297
NRS score, mean ± sd	0.44 ± 0.69	0.60 ± 1.10	0.293
Hemoglobin, mean ± sd	85.14 ± 17.88	82.43 ± 21.86	0.341
Platelet count, mean ± sd	188.84 ± 72.24	177.96 ± 82.77	0.330
PNI, mean ± sd	41.99 ± 4.97	37.04 ± 6.00	1.553e−09
GNRI, mean ± sd	98.65 ± 10.07	92.42 ± 10.90	6.071e−05
Surgical duration (minutes), median (IQR)	36.00 (30.00, 50.00)	50.00 (32.00, 54.00)	0.002
Intraoperative bleeding volume (ml), median (IQR)	10.00 (9.00, 30.00)	30.00 (10.00, 32.00)	0.002
Surgical incision length (cm), mean ± sd	2.83 ± 0.82	3.28 ± 0.93	0.000
Postoperative hospitalization time (days), mean ± sd	6.59 ± 3.55	7.09 ± 3.75	0.351
Total hospitalization time (days), mean ± sd	12.42 ± 3.02	13.12 ± 2.78	0.107
Surgical cost (yuan), mean ± sd	1137.60 ± 1114.20	1139.80 ± 1013.30	0.989
Total hospitalization expenses (yuan), mean ± sd	1.71 ± 6550.10	1.77 ± 5536.40	0.513
Surgical cost/total cost (%), mean ± sd	0.07 ± 0.06	0.06 ± 0.03	0.385
Previous history of abdominal surgery, *n* (%)			0.711
No	129.00 (88.40%)	58.00 (86.60%)	
Yes	17.00 (11.60%)	9.00 (13.40%)	
Primary disease (0: chronic nephritis 1: diabetes nephropathy 2: hypertensive nephropathy 3: others), *n* (%)			0.047
3	102.00 (69.40%)	41.00 (61.20%)	
0	8.00 (5.40%)	9.00 (13.40%)	
1	36.00 (24.50%)	14.00 (20.90%)	
2	1.00 (0.70%)	3.00 (4.50%)	
Catheter flow dysfunction, *n* (%)			1.000
No	139.00 (94.60%)	64.00 (95.50%)	
Yes	8.00 (5.40%)	3.00 (4.50%)	
Dialysate leakage, *n* (%)			0.579
No	144.00 (98.00%)	64.00 (95.50%)	
Yes	3.00 (2.00%)	3.00 (4.50%)	
Catheter migration, *n* (%)			0.798
No	143.00 (97.30%)	64.00 (95.50%)	
Yes	4.00 (2.70%)	3.00 (4.50%)	
Exit-site infection (ESI), *n* (%)			2.199e−20
No	147.00 (100.00%)	34.00 (50.70%)	
Yes	0.00 (0.00%)	33.00 (49.30%)	
Abdominal catheter tunnel infection, *n* (%)			0.001
No	147.00 (100.00%)	61.00 (91.00%)	
Yes	0.00 (0.00%)	6.00 (9.00%)	

### 3.2 Independent risk factors for long-term infection in PD

**FIGURE 2:**
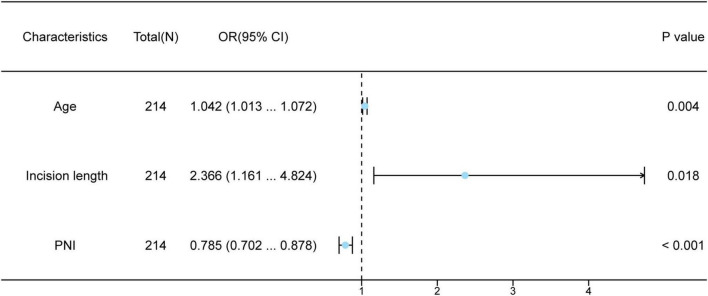
Multivariate logistic regression analysis of patients with infections undergoing peritoneal dialysis.

Univariate and multivariate logistic regression analyses were executed in both groups (Table 2). Univariate logistic regression analysis indicated that patient age, operative time for PD catheterization, surgical incision length, intraoperative blood loss, PNI, and GPNI emerged as independent potential risk indicators for infection in patients undergoing PD (all *p* < 0.05). We threw these variables into a multivariate logistic regression analysis to pick out independent factors related to long-term infections in these patients, including age, surgical incision length and PNI. A random forest plot was developed to further assess the precision and significance of these predictive variables. As is shown in Figure 2, increasing patient age (OR = 1.042, 95% CI: 1.013–1.072, *p* = 0.004), longer surgical incision length (OR = 2.366, 95% CI: 1.161–4.824, *p* = 0.018), in addition, lower PNI (OR = 0.785, 95% CI: 0.702–0.878, *p* < 0.001) were elements that increase susceptibility for long-term infection in patients undergoing PD.

**TABLE 2:** Univariable and multivariable logistic regression analysis of patients with infections undergoing peritoneal dialysis.

	Univariate analysis			Multivariate analysis	
**Characteristics**	**Total (*N*)**	**OR (95% CI) Univariate analysis**	***P*-value**	**OR (95% CI) Multivariate analysis**	***P*-value**
Age	214	1.034 (1.010–1.059)	0.005	1.042 (1.013–1.072)	0.004
Gender	214				
Female	80	Reference			
Male	134	1.621 (0.874–3.007)	0.126		
Height	214	1.003 (0.981–1.025)	0.811		
Weight	214	0.999 (0.978–1.020)	0.910		
BMI	214	1.047 (0.968–1.133)	0.247		
Serum creatinine	214	1.000 (0.999–1.001)	0.628		
Blood urea nitrogen	214	0.996 (0.986–1.007)	0.511		
Serum sodium	214	1.004 (0.919–1.096)	0.934		
Serum potassium	214	0.993 (0.955–1.031)	0.703		
Serum calcium	214	0.897 (0.306–2.631)	0.843		
Serum phosphorus	214	0.974 (0.874–1.086)	0.637		
Serum albumin	214	0.831 (0.775–0.890)	<0.001	0.817 (0.741–0.900)	0.068
Blood PTH	214	1.000 (1.000–1.001)	0.251		
Total cholesterol (mmol)	214	0.913 (0.720–1.157)	0.452		
White blood cell count (× 10^9^)	214	0.987 (0.882–1.104)	0.814		
Neutrophil count (10^9^)	214	0.997 (0.885–1.122)	0.956		
Lymphocyte count (10^9^)	214	0.726 (0.398–1.324)	0.296		
Hemoglobin	214	0.993 (0.978–1.008)	0.340		
NRS score	214	1.230 (0.885–1.709)	0.217		
Platelets	214	0.998 (0.994–1.002)	0.330		
PNI	214	0.841 (0.789–0.896)	<0.001	0.785 (0.702–0.878)	<0.001
GNRI	214	0.941 (0.911–0.971)	<0.001	1.011 (0.967–1.058)	0.622
Operation time (minutes)	214	1.042 (1.015–1.071)	0.002	1.033 (0.940–1.136)	0.498
Intraoperative blood loss (ml)	214	1.042 (1.015–1.070)	0.002	1.073 (0.880–1.307)	0.486
Surgical incision length (cm)	214	1.834 (1.293–2.601)	<0.001	2.366 (1.161–4.824)	0.018
Postoperative hospitalization time (days)	214	1.038 (0.960–1.124)	0.350		
Total hospitalization time (days)	214	1.085 (0.982–1.198)	0.108		
Surgical cost (yuan)	214	1.000 (1.000–1.000)	0.989		
Total hospitalization expenses (yuan)	214	1.000 (1.000–1.000)	0.511		
Surgical cost/total cost (%)	214	0.051 (0.000–44.688)	0.389		
Previous history of abdominal surgery	214				
No	188	Reference			
Yes	26	1.177 (0.496–2.797)	0.711		
Primary disease	214				
Chronic nephritis	17	Reference		Reference	
Diabetes nephropathy	50	0.346 (0.111–1.075)	0.067	0.310 (0.085–1.132)	0.076
Hypertensive nephropathy	4	2.667 (0.229–31.069)	0.434	2.372 (0.181–31.022)	0.510
Others	143	0.357 (0.129–0.990)	0.048	0.358 (0.108–1.191)	0.094
Surgical approach	214				
Laparoscopy	14	Reference		Reference	
Laparotomy	100	4.345 (0.923–20.444)	0.063	0.835 (0.006–119.240)	0.943
Percutaneous puncture	100	1.792 (0.374–8.595)	0.466	7.716 (0.859–69.314)	0.068

### 3.3 Development and analysis of the nomogram model

**FIGURE 3:**
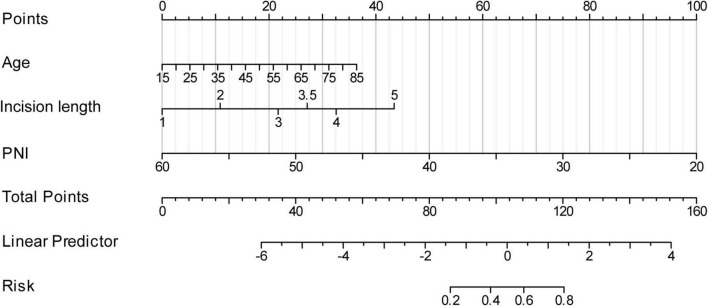
Establishment of the nomogram for predicting infections in patients undergoing peritoneal dialysis.

**FIGURE 4:**
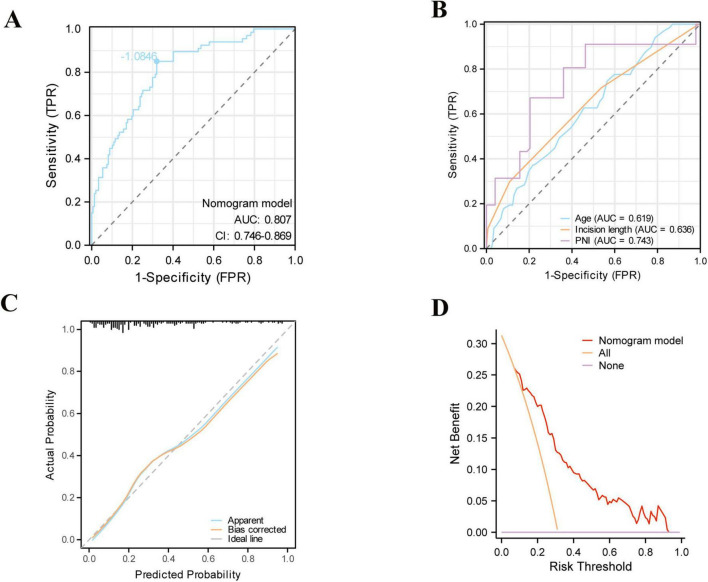
Evaluation of the nomogram. **(A)** Area under the ROC curves (AUC) for the prediction of infections using the nomogram in patients undergoing peritoneal dialysis. **(B)** AUC for the prediction of infections using single indicator. **(C)** The calibration curve for the risk of infections. **(D)** Decision curve analysis (DCA) of the nomogram (red line).

As illustrated in Figure 3, based on the three independent predictive factors obtained from multivariable logistic regression, the nomogram model was rigorously formulated to predict infection risk in PD individuals. The results indicated that a PNI score of 20 corresponded to the highest risk score (100), followed by an incision length of 5 cm (43 points). When the risk factors for infections in patients are visualized, individual risks for developing long-term infections can be predicted. First, each independent risk factor contributing to infections in patients undergoing PD was projected onto the first row of the scale to derive scores for each factor, and by adding up the values of all 3 risk factors, total score was calculated. A higher total score indicates a higher risk of infection for these patients. The research sample was used as the training set, with 1,000 repeated samplings performed, and internal validation was conducted using five-fold cross-validation. As shown in Figure 4A, the AUC value was 0.807 (95% CI: 0.746–0.869), indicating that the model demonstrated good discriminative accuracy in predicting the risk of long-term infection in PD patients. The corresponding AUC values for PNI, surgical incision length, and age were 0.743, 0.636, and 0.619. Specifically, PNI exhibited higher predictive accuracy compared to surgical incision length and age (Figure 4B). To evaluate the validity of the modeling results, a calibration plot for the infection risk prediction model in PD individuals was generated. Figure 4C showed that the observed outcomes were in high agreement with the predicted outcomes. DCA was performed to assess its clinical utility. As illustrated in Figure 4D, according to the decision curve, when the threshold probability for a specific patient ranged between 14 and 92%, the net benefit of this predictive model (red curve) was consistently higher than that of the “All” and “None” lines, demonstrating its practical value.

## 4 Discussion

The latest statistics released by the Chinese Center for Disease Control and Prevention in 2023 indicate that approximately 82 million adults in China suffer from chronic kidney disease (CKD). As renal function deteriorates in patients suffering from CKD, the condition may eventually progress to an irreversible phase known as ESRD, which necessitates ongoing renal replacement therapy, like dialysis or a kidney transplant, to maintain life. PD, a treatment method that prolongs the lives of patients with ESRD, helps preserve residual kidney function and improves their quality of life, contributing to its increased prevalence among patients with ESRD. Research suggests that over 272,000 patients worldwide are currently treated with PD (13, 14). However, PD-related infections remain a significant threat to patient safety, potentially leading to catheter removal, changes in dialysis methods, or even life-threatening situations. Being one of the most frequently encountered complications, infection is the primary factor leading to the efficacy of dialysis (15). Infections occurring during PD can result in treatment failure and pose life-threatening risks. Consequently, it is of great significance and value for medical clinicians to rapidly and accurately detect the individuals who are at a high risk level, implement timely preventive measures targeting related risk factors, reduce infection rates, and improve patient prognosis and living standards (16).

This study show that older age, longer PD catheterization incision lengths, and lower PNI are risk factors for infections in patients undergoing PD. Among these, PNI had the most significant impact on infection occurrence compared to age and incision length. PNI is a composite index that assesses the immune, inflammatory, and nutritional status of patients (17), and is worked out rooted in serum albumin and peripheral blood lymphocytes. It is widely used in prognostic assessments for various conditions, including cancer and cardiovascular diseases (18–20). Previous research has brought to light that PNI takes on a major role in predicting outcomes for patients undergoing PD (21, 22). Although preliminary small-scale studies have suggested an association between low PNI and the incidence of peritonitis in PD individuals, multicenter validation remains lacking, and a low PNI is key to predicting mortality in this population (23), which is consistent with the predictive directions of this study. Patients with ESRD undergoing long-term dialysis often experience malnutrition, largely due to dietary restrictions (low-protein diets), nutrient loss during dialysis, and increased catabolic metabolism due to inflammatory cytokines. The incidence of diseases stemming from malnutrition greatly affects patients’ health and well-being, and increases the risk of infection in individuals undergoing PD (24, 25). Low nutritional status in these patients, coupled with anemia and hypoalbuminemia, creates a vicious cycle that exacerbates infection risk (26). So far, the criterion of PNI in the PD population has not been standardized, and whether interfering with the PNI factor can reduce the probability of infection in PD patients still requires further validation through large-sample, multicenter studies.

According to the latest data from the United States Renal Data System published in 2023, the highest incidence of ESRD is seen in individuals over 75 years of age. Older patients undergoing dialysis often have weakened immune systems and multisystem diseases, making them more susceptible to infections. Relevant studies have confirmed that older patients undergoing PD are more likely to develop infections than younger patients (27). Additionally, patients with ESRD over 60 years of age experience declining immune function, diminished quantity and reduced immune cell efficacy, and damage to the peritoneum caused by prolonged dialysis, often alongside ongoing health issues like diabetes, hypertension, and hemodynamic diseases. This results in reduced resistance to various pathogens, thereby increasing the likelihood of infections (28). A single-centre retrospective study also revealed that older patients had higher rates of peritonitis and associated mortality (29).

The length of the incision used for PD catheterization is another contributing factor. Historically, open surgical methods were predominantly used for PD catheterization. However, open catheterization causes greater trauma to patients, with longer incisions significantly affecting postoperative recovery (30). For obese patients with ESRD, larger surgical incisions increase the risk of fat liquefaction and infection during recovery (31). Additionally, larger incisions provide a greater area for direct contact with the abdominal cavity. If sterile protocols are violated during surgery or postoperative care is inadequate, infections may occur, disrupting normal dialysis processes.

In summary, infections in patients undergoing PD are influenced by multiple factors including individual differences, treatment environments, and operational techniques. Predictive models cannot capture all these factors, limiting their generalizability across different patient populations or treatment contexts. Furthermore, another limitation of this study is that we did not investigate the types of pathogens (e.g., gram-positive and gram-negative bacteria) and the sites of infection (e.g., exit-site infection vs. peritonitis). Additionally, most current research focuses on constructing individualized predictive models within single centres, limiting their clinical applicability and accuracy. Further exploration is needed to optimize predictive models and establish personalized treatment plans that improve prognosis and quality of life for patients undergoing PD.

## 5 Conclusion

In conclusion, this study identified patient age, surgical incision length, and PNI as individual risk determinants for infections in patients receiving PD, which were used to construct a nomogram model. This model demonstrated good predictive efficacy for long-term infections in patients undergoing PD. Moreover, it can be employed as a valuable reference for clinical staff to detect high-risk individuals at an early stage, facilitating prompt interventions to reduce infection rates and improve outcomes.

## Data Availability

The original contributions presented in this study are included in this article/Supplementary material, further inquiries can be directed to the corresponding authors.
